# Supplementary material for: Neuroprotection in Glaucoma: Basic Aspects and Clinical Relevance
Source: J Pers Med. 2022 Nov 10;12(11):1884. doi: 10.3390/jpm12111884 (PMC9697907; doi:10.3390/jpm12111884)
Supplement: Supplementary file 1 [file jpm-12-01884-s001.zip › jpm-1950378-supplementary.pdf]

Supplementary Table S1. Summary table of the included clinical studies								
**Modality**	**Proposed Mechanism**	**Study**	**Country**	**Study Design**	**Intervention**	**Sample Size**	**Key Findings**	**Clinical Implications**
NGF	1. Promote differentiation, survival, and growth of neurons<br>2. Prevent RGCs and axons from degeneration and inhibit apoptosis	Lambiase et al, 2019	Italy	Clinical trial	Topical 200 µg/mL NGF	3 patients with advanced and progressive glaucoma	1. Improved inner retinal and visual pathway function<br>2. Improved visual field MD, contrast sensitivity, and VA	1. Topical NGF has the potential in improving visual function in patients with severe glaucoma
		Beykin et al, 2022	United States	Prospective, single-center, randomized, double-masked, vehicle-controlled, parallel-group study.	Topical 180 µg/mL recombinant human NGF for 8 weeks	60 POAG (40 treatment; 20 control)	1. No serious adverse effect<br>2. No difference in visual field or RNFL thickness	1. Topical 180 µg/mL recombinant human NGF is tolerable and safe<br>2. Clinical efficacy remains to be justified
BDNF	1. Activation of extracellular signal-regulated kinases 1/2 and c-jun,<br>2. Inhibition of caspase 2	Oddone et al, 2017	Italy	Observational study	NA	45 POAG / 15 healthy control	1. Serum levels of BDNF and NGF were lower in the glaucoma group, particularly in early and moderate glaucoma	1. Serum levels of BDNF and NGF may serve as biomarkers for the early detection of glaucoma

		Uzel et al, 2018	Turkey	Prospective, cross-sectional study	Trabeculectomy	20 POAG / 19 healthy control	1. Pre-operative serum and AH levels of BDNF were lower in the glaucoma groups<br>2. Serum levels of BDNF increased three months after trabeculectomy	1. Reduction of the IOP after trabeculectomy can increase BDNF levels.
		Cha et al, 2021	Korea	Prospective, observational study	NA	30 POAG / 30 NTG / 30 healthy control	1. Serum and AH levels of BDNF were lower in the glaucoma groups<br>2. Serum BDNF levels were lower in patients with NTG than in those with POAG	1. Serum levels of BDNF may serve as a biomarker for NTG and POAG
CNTF	1. Promote axonal regeneration<br>2. Stimulate the survival of developing neurons	Shpak et al, 2017	Russia	Observational study	NA	55 POAG / 29 healthy control	1. AH and lacrimal fluid levels of CNTF were lower in the glaucoma group.<br>2. The decrease of CNTF levels in	1. Lacrimal fluid and AH levels of CNTF may serve as biomarkers for POAG<br>2. Visual field may be indirectly used

							AH and lacrimal fluid were more prominent in severe glaucoma<br>3. AH levels of CNTF correlated with VFI	to estimate AH levels of CNTF
Ginkgo Biloba	1. Increased blood flow<br>2. Reduce oxidative stress in the mitochondria and scavenge free radicals	Quaranta et al, 2003	Italy	Prospective randomized placebo-controlled study	Oral 40 mg GBE 3 times daily for 4 weeks, followed by 8 weeks of a wash-out, then 4 weeks of placebo tablets; 2 groups crossover	27 NTG	1. Improved MD and pattern standard deviation after GBE treatment	1. GBE can improve preexisting visual field defects in patients with NTG
		Lee et al, 2013	Korea	Retrospective study	Oral 80 mg GBE 2 times daily for an unknown period	42 NTG	1. Significant improvement in regression coefficients of MD, PSD, and VFI<br>2. The regression coefficient of mean total deviation change	1. GBE can slow visual field damage in patients with NTG

							increased after the use of GBE	
		Guo et al, 2014	China	Prospective randomized placebo-controlled study	Oral 40 mg GBE 3 times daily for 4 weeks, followed by 8 weeks of a wash-out, then 4 weeks of placebo tablets; 2 groups crossover	35 NTG (newly diagnosed)	1. No significant change in MD and contrast sensitivity	1. GBE does not affect visual field or contrast sensitivity in newly-diagnosed NTG patients
		Sabaner et al, 2021	Turkey	Prospective study	Oral 120 mg GBE once a day for 4 weeks	60 healthy volunteers	1. Significant increase in peripapillary and superior, inferior, temporal quadrant radial peripapillary capillary vascular density after GBE consumption	1. GBE can lead to vascular morphological changes in the radial peripapillary capillary.<br>2. The functional correlation remains to be justified.
Brimonidine	1. Increase NTFs<br>2. Alter NMDA receptors	Evans et al, 2003	United States / United Kingdom	Randomized double-blind study	Monotherapy with either brimonidine 0.2%	16 POAG	1. Patients treated with brimonidine had improved	1. Brimonidine can improve contrast sensitivity 3

	3. Boost BDNF expression in RGCs<br>4. Interfere with the amyloid-β pathway and lower its levels				or timolol gel 0.5%		contrast sensitivity<br>2. Similar IOP reduction in both groups	months after administration
		Tsai et al, 2005	Taiwan	Prospective comparative unmasked study	Monotherapy with either brimonidine 0.2% or timolol maleate 0.5% for 12 months	39 POAG (19 brimonidine; 20 timolol)	1. RNFL thickness decreased in patients receiving timolol while it remained unchanged in the brimonidine group<br>2. Similar IOP reduction between groups	1. Brimonidine may lead to less RNFL change than timolol
		Krupin et al, 2011	United States	Randomized controlled study	Twice-daily monotherapy with either brimonidine or timolol	178 NTG (99 brimonidine; 79 timolol)	1. Patients treated with brimonidine had less visual field progression by pointwise linear regression<br>2. Mean IOP was identical between 2 groups at all times	1. Brimonidine may have an effect to slow visual field progression, independent of IOP

Calcium channel blocker	1. prevent RGC death caused by calcium influx<br>2. Increase local blood flow in ischemic tissues by inducing vasodilation	Sawada et al, 1996	Japan	Prospective study	Brovincamine fumarate 20 mg 3 times daily or placebo 3 times daily for at least 2.5 years	28 NTG (14 treatment and 14 control)	1. Six out of 14 treated patients showed visual field improvement whereas none of the control group had improvement in the visual field	1. Brovincamine may have a favorable effect on some NTG patients.
		Koseki et al, 1999	Japan	Prospective study	Brovincamine fumarate 20 mg 3 times daily or placebo 3 times daily for 2 years	52 NTG	1. The slope of change in MD was less negative in the brovincamine group	1. Brovincamine may slow visual field progression in NTG patients
		Koseki et al, 2008	Japan	Randomized, placebo-controlled, double-masked, single-center trial	Oral nilvadipine 2 mg twice daily for 3 years	33 OAG (17 treatment and 16 control)	1. The slope of change in MD was less negative in the nilvadipine group<br>2. Quantitative indexes of circulation in the optic disc rim and choroid in the foveal area	1. Nilvadipine may slow visual field progression and increase blood circulation around the optic disc and foveal area

							remained increased compared with baseline measurements in the nilvadipine group	
		Hu et al, 2021	China	Retrospective study	Oral nimodipine 60 mg for 3 months	20 NTG	1. Parafoveal vessel density significantly increased while peripapillary capillary vessel density remained unchanged at 3 months after the use of nimodipine<br>2. The parafoveal vessel density significantly correlated with the administration of nimodipine, RNFL thickness, and MD	1. Nimodipine can lead to vascular morphological changes in the macula, which correlated positively with structural and functional parameters in glaucoma evaluation

		Duan et al, 2022	China	Retrospective study	Combined therapy with nimodipine plus latanoprost versus monotherapy of latanoprost	87 POAG (46 combined; 41 monotherapy)	1. Ocular hemodynamics, visual field defects, 24-hour peak IOP, binocular optic disc parameters, and life quality were better in the combined treatment group.<br>2. No difference in adverse effects between groups	1. Nimodipine combined with latanoprost is effective and safe in treating POAG patients
Memantine	1. Inhibit excessive glutamate activity	Weinreb et al, 2018	United States	Randomized, double-masked, placebo-controlled, parallel-group, multicenter study	Memantine 20 mg, memantine 10 mg, or placebo for 48 months	2298 OAG	1. Treatment with memantine 10 mg and 20 mg did not affect retarding visual field progression	1. Daily treatment with oral memantine 10 mg or 20 mg for 48 months is not effective to prevent visual field progression
Citicoline	1. Reduce glutamate excitotoxicity,	Parisi et al, 1999	Italy	Randomized study	Citicoline 1000 mg/day intramuscularly	40 OAG (25 treatment; 15 control)	1. Treatment with citicoline leads to improvement of	1. Intramuscular injection of citicoline may improve retinal

	2. Lower oxidative stress in RGC damage<br>3. Improve axonal transport deficit						VEP and PERG parameters	and visual pathway function
		Ottobelli et al, 2013	Italy	Prospective study	Citicoline in oral solution for 2 years	41 POAG	1. The rate of visual field progression decreased significantly after the treatment	1. Oral solution of citicoline may slow glaucoma progression
		Parisi et al, 2015	Italy	Prospective randomized masked study	Topical citicoline (OMK1) 3 times daily versus beta-blocker monotherapy	47 OAG (24 citicoline; 23 beta-blocker)	1. Increasing PERG P50-N95 and VEP N75-P100 amplitudes, and shortening of VEP P100 implicit times after topical citicoline<br>2. No changes in PERG and VEP in the beta-blocker group	1. Topical citicoline can induce enhancement in retinal and visual cortical function
		Rossetti et al, 2020	Italy	Randomized, double-masked, placebo-controlled, multicenter study	Topical citicoline 3 times daily for 3 years	80 OAG (40 citicoline; 40 control)	1. Better rates of progression for 10-2 MD in the treated group	1. Citicoline may prevent functional and structural progression in

							2. Less loss of RNFL in the treated group	patients with controlled IOP
Antioxidant Q10	1. Delay RGC apoptosis<br>2. Reduce glutamate concentration	Parisi et al, 2014	Italy	Retrospective study	Coqun (coenzyme Q10 and vitamin E, 2 drops daily) plus beta-blocker versus beta-blocker monotherapy	43 OAG (22 combined; 21 beta-blocker monotherapy)	1. PERG P50 and VEP P100 implicit times were decreased, whereas PERG P50-N95 and VEP N75-P100 amplitudes were increased	1. The addition of Coqun can improve retinal and visual cortical function
		Ozates et al, 2019	Turkey	Prospective randomized study	Coqun (coenzyme Q10 and vitamin E, 2 drops daily) for 1 month	47 pseudo-exfoliative glaucoma (23 treatment; 24 control), 17 pseudo-exfoliation syndrome	1. Mean AH superoxide dismutase level was lower in the pseudo-exfoliative glaucoma + Coqun group than in the pseudo-exfoliative glaucoma group	1. Topical Coqun may decrease superoxide dismutase, an oxidative stress marker, in AH

Nicotinamide (vitamin B3)	1. prevents age-related declines in nicotinamide adenine dinucleotide	Nzoughet et al, 2019	France	Observational study	NA	34 POAG / 30 control	1. Plasma level of nicotinamide was lower in POAG patients	1. Glaucoma is associated with lower plasma levels of nicotinamide
		Hui et al, 2020	Australia	Randomized, double-masked, crossover study	nicotinamide 1.5 g/day for 6 weeks, then 3.0 g/day for 6 weeks followed by crossover with placebo without washout	49 glaucoma (26 nicotinamide first; 23 placeboes first)	1. Saturated photopic negative response amplitude improved more following the consumption of nicotinamide<br>2. Improvement of visual field MD ≥1 dB and less deterioration were observed following nicotinamide treatment	1. Oral nicotinamide can lead to improvement in inner retinal function in glaucoma patients
		De Moraes et al, 2022	United States	Randomized, double-blind, placebo-controlled study	Increasing oral doses of nicotinamide (1000 to 3000 mg) and pyruvate	32 OAG (21 treatments; 11 placeboes)	1. A greater number of improving visual field test locations and improved rates of	1. High doses of nicotinamide supplementation can lead to short-term

					(1500 to 3000 mg) versus placebo		change of PSD in the treated group	improvement in visual field function
Statins	1. Improve survival of RGC<br>2. Reduce apoptosis<br>3. Suppress glial activation in the retina	De Castro et al, 2007	United States	Retrospective cohort study	Statins only, aspirin only, statins plus aspirin, control	76 OAG suspect (12 statins only; 13 aspirin only; 12 statins plus aspirin; 39 control)	1. Statins only group exhibited slower progression of rim volume, RNFL cross-sectional area, and mean global RNFL thickness than the control	1. Statins may be associated with optic nerve structural parameters in glaucoma suspects
		Kang et al, 2022	United States	Retrospective cohort study	History of statins use	1978 POAG and glaucoma suspect (775 subjects had statins use)	1. Similar rates of change for MD and RNFL thickness between the two groups<br>2. Multivariable models revealed no association between the duration of statins use and rates of MD or RNFL thickness change	1. History of statins use or duration of statins use is not associated with structural or functional parameter changes in patients with glaucoma or glaucoma suspect

Stem cell therapy	1. Regenerate RGCs and produce new cells of different kinds.<br>2. Provide a favorable neurotrophic environment to the damaged RGCs	Vivela et al, 2021	Brazil	Clinical trial	A single intravitreal injection of autologous mesenchymal stem cells	2 OAG in advanced stage	1. No improvement in VA or visual field sensitivities<br>2. ERG was stable for 1 year in one case and 2 weeks in another case due to the development of retinal detachment	1. Mesenchymal stem cell therapy seems to be capable of maintaining ERG in severe glaucoma cases<br>2. The risk of severe adverse effects warrants further investigation and modification

AH, aqueous humor; BDNF, brain-derived neurotrophic factor; CNTF, ciliary neurotrophic factor; GBE, ginkgo biloba; IOP, intraocular pressure; MD, mean deviation; NA, not available; NGF, nerve growth factor; NMDA, N-methyl-D-aspartate; NTG, normal tension glaucoma; NTFs, neurotrophic factors; PERG, pattern electroretinogram; POAG, primary open-angle glaucoma; PSD, pattern standard deviation; RGCs, retinal ganglion cells; RNFL, retinal nerve fiber layer; VA, visual acuity; VEP, visual evoked potential; VFI, visual field index
